# Veterinary College

**Published:** 1856-05

**Authors:** 


*Veterinary College.*—A bill has been introduced in the New York Legislature to incorporate the New York College of Veteriuary Surgeons of the City of New York. The corporators are William Cooper, H. Williams, M. D., John Lockwood, Thomas D. Andrews, M. D., J. Ogle, M. D., T. Nortram, T. Grice and P. Green. The object is to promote veterinary science and instruction in the department of learning connected therewith. It allows them to hold and convey real estate to the amount of $100,000. It gives power to the trustees to confer the degree of V. S. (Veterinary Surgeon) on any man of the age of 21 years, who may have studied three years with some Veterinary Surgeon duly licensed, and have attended two complete courses of lectures, one of which shall have been delivered by the Professors of said College.—*N. Y. Daily Times.*

